# Knowing the Enemy Is Halfway towards Victory: A Scoping Review on Opioid-Induced Hyperalgesia

**DOI:** 10.3390/jcm11206161

**Published:** 2022-10-19

**Authors:** Tiago J. Sampaio-Cunha, Isabel Martins

**Affiliations:** 1 Department of Biomedicine, Unit of Experimental Biology, Faculty of Medicine of the University of Porto, 4200-319 Porto, Portugal; 2 i3S–Institute for Research & Innovation in Health, University of Porto, 4200-135 Porto, Portugal; 3 IBMC-Institute for Molecular and Cell Biology, University of Porto, 4200-135 Porto, Portugal

**Keywords:** opioids, opioid-induced hyperalgesia, neural-inflammatory crosstalk, µ-opioid receptor, opioid signaling, alternative splicing

## Abstract

Opioid-induced hyperalgesia (OIH) is a paradoxical effect of opioids that is not consensually recognized in clinical settings. We conducted a revision of clinical and preclinical studies and discuss them side by side to provide an updated and renewed view on OIH. We critically analyze data on the human manifestations of OIH in the context of chronic and post-operative pain. We also discuss how, in the context of cancer pain, though there are no direct evidence of OIH, several inherent conditions to the tumor and chemotherapy provide a substrate for the development of OIH. The review of the clinical data, namely in what concerns the strategies to counter OIH, emphasizes how much OIH rely mechanistically on the existence of µ-opioid receptor (MOR) signaling through opposite, inhibitory/antinociceptive and excitatory/pronociceptive, pathways. The rationale for the maladaptive excitatory signaling of opioids is provided by the emerging growing information on the functional role of alternative splicing and heteromerization of MOR. The crossroads between opioids and neuroinflammation also play a major role in OIH. The latest pre-clinical data in this field brings new insights to new and promising therapeutic targets to address OIH. In conclusion, although OIH remains insufficiently recognized in clinical practice, the appropriate diagnosis can turn it into a treatable pain disorder. Therefore, in times of scarce alternatives to opioids to treat pain, mainly unmanageable chronic pain, increased knowledge and recognition of OIH, likely represent the first steps towards safer and efficient use of opioids as analgesics.

## 1. Introduction

Opioid-induced hyperalgesia (OIH) is defined as abnormally increased nociception in consequence of opioid exposure. Opioid-induced effects on the nociceptive system may be conceptualized as the sum of the following properties: (1) a relative block in nociceptive transmission (responsible for analgesia), (2) sensitization of the system towards afferent nociception (the exacerbation of which explains OIH) and (3) desensitization of the system towards opioid-agonists (the property underlying tolerance) [1,2]. OIH is an established phenomenon on animal studies [2,3]. It’s clearly observable and documentable on rodents, nevertheless, serious doubts appear when attempting to translate this phenomenon to humans [4,5,6,7].

One of the reasons that explains why OIH is questioned as a clinically relevant mechanism is the lack of a serious estimate of its prevalence, transverse to all medical conditions in which it may manifest, that is, conditions in which opioids are used for analgesia [1,4,6]. This is due to the lack of robust clinical studies evaluating the impact of this phenomenon. Another problem hindering the recognition of OIH arises from the clinical similarity between OIH and tolerance, which may impair OIH diagnosis. Nonetheless, OIH and tolerance are differential diagnoses [1,6]. In case of clinical suspicion of OIH, a therapeutic trial involving the manipulation of the opioid dose can establish the definitive diagnosis, as an OIH patient is expected to improve when lowering the dose and worsen when increasing the dose. In the case of opioid tolerance, a patient placed on a low-dose opioid regimen will have its pain worse and require an increased dose for pain relief [5,8]. This distinction between OIH and opioid tolerance was also established in human volunteer studies, where it was possible to control opioid administration and to measure changes in pain thresholds quantitatively [3,9,10]. Thus far, OIH assessment has been performed by using different experimental models such as noxious thermal (hot and cold) and electrical stimuli. However, these experimental pain models have been shown to produced different results [11]. Therefore, implementing consensual experimental pain models, clinically relevant, would contribute to establishing the clinical diagnosis of OIH [8]. The dose regimen at which opioids induce OIH is variable. A bimodal hyperalgic- vs. analgesic effect of MOR activation is well documented, both in rodent studies [12] and in humans [13,14]; whereby normal doses of opioid antagonists cause hyperalgesia, while micro- and low-doses have analgesic effects. At low doses, opioid antagonists are now known to block the co-existing excitatory signaling of mu-opioid receptors (MOR) through a mechanisms that entails the interaction of the antagonists with the MOR-interacting scaffolding protein filamin A, rather than via a direct interaction with MOR which occurs at normal and higher doses [15,16]. Opioid agonists exert both inhibitory and excitatory effects through G protein coupling, the latter being generally masked by the inhibitory effects produced by opioids at concentrations required to produce analgesia [15]. Low-dose hyperalgesia induced by opioids has also been observed in rodents. Though less numerous, human cases of OIH have also been diagnosed in patients chronically treated with low-dose and normal-doses of opioids [5,6,7,11]. Nonetheless, the increase in opioid dose appears suggestive of greater propensity to induce OIH. For example, very-high-dose acute opioid administrations (“promoted by a single event”, such as surgery) have been observed to cause OIH in humans.

The lack of recognition of OIH by clinicians, in part due to the lack of awareness [6,7], has serious consequences to chronic pain patients. First and foremost, it implies the underdiagnosis and undertreatment of addressable pain conditions. In fact, when pain is worse after raising opioid doses, not considering the hypothesis of OIH, physicians would continue to prescribe increased doses of opioids, as it would be the practice for the clinical entity of opioid tolerance. Thus, the objective of this review is to place, side-by-side, clinical studies registering the occurrence of OIH and the latest preclinical studies to provide an enlightened perspective on OIH evidence and raise awareness on how to address and prevent it.

## 2. Materials and Methods

**Figure 1:**
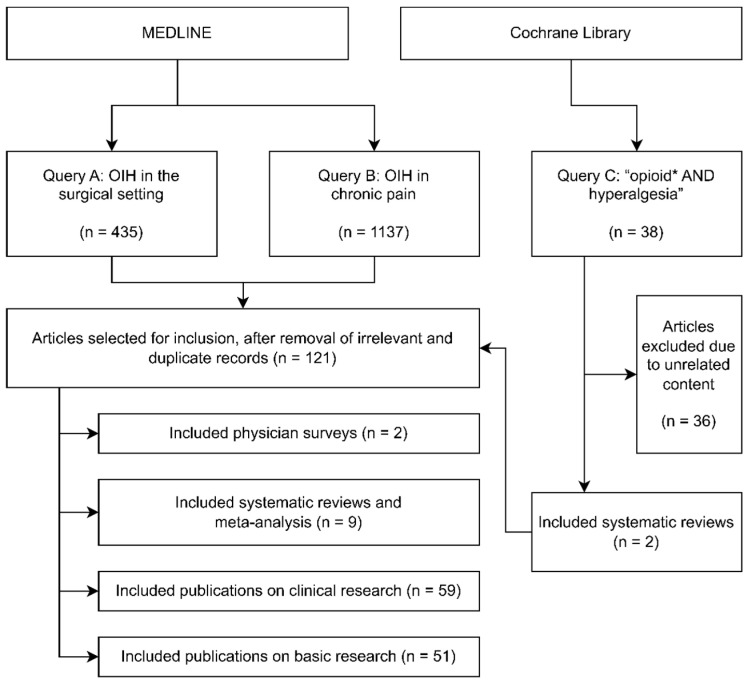
Flowchart depicting the selection of relevant literature.

Articles eligible for consideration in this review were identified through a search in the electronic databases of MEDLINE and the Cochrane Library. The search was made until the 31st of March of 2022, using several queries, organized by sub-theme. Peer reviewed original articles and systematic reviews published until the last date of the search were included. The full electronic search strategy conducted in MEDLINE and the Cochrane Library is depicted in Figure 1. Regarding OIH in the surgical setting, the following MEDLINE query was searched: “((OIH or “opioid induced hyperalgesia”) and (operative or surgery or surgical)) or (opioid* and surg* and hyperalgesia and patient*)”; from this search a total of 435 articles were shown and analyzed. Regarding OIH in chronic pain the following MEDLINE query was searched: (((“low back pain” OR “lumbar pain”) OR (neuropathic) OR (chronic AND patient *) OR (fibromyalgia) OR (migraine)) AND hyperalgesia AND opioid *); from this search a total of 1137 articles were shown and analyzed. Furthermore, search by forwards and backwards referencing was performed where appropriate.

In the Cochrane Library, the keywords “opioid * AND hyperalgesia” were used in “All Text”; from this search, 38 systematic reviews were shown and analyzed.The first author manually scanned titles and abstracts and then the full texts of the publications identified by the searches performed in MEDLINE and the Chochrane Library. The articles were selected for inclusion after discussion with the second author. A total of 121 articles were selected for inclusion in this review. Included literature contained: (I) physician-based studies, with large-scale survey-based methodologies (n = 2); (II) systematic reviews with meta-analysis that included observational and experimental clinical studies of analgesic modalities containing relevant data for clinical identification of OIH (n = 9); (III) other observational and experimental clinical studies, as well as clinical case-reports, assessing the impact of different analgesic modalities that provided relevant data for clinical identification and characterization of OIH and modulation of hyperalgesia through therapeutic actions (like change in opioid substance and/or dose and/or the addition of adjuvant medication) (n = 59); (IV) pre-clinical molecular, cell-based and animal studies, original articles as well as reviews, describing biological basis of OIH (n = 51).Pre-clinical data from original research articles as well as from review articles were selected based on their relevance to explain the OIH pathophysiology as clinically observed.The clinical data were presented in three tables summarizing the characteristics of the clinical original studies analyzed in the context of chronic pain, in the surgical context, also summarizing the outcomes of all meta-analysis performed in the surgical context. The variables and effects measured in the clinical studies were discussed throughout the text, considering the knowledge gathered by the pre-clinical studies selected and included in this review.

## 3. Results

### 3.1. OIH in Chronic Pain

#### 3.1.1. Opioid Prescription and Epidemiology

Prescription of opioids varies considerably between countries and within continents. In North America, the United States and Canada face a problem of over-prescription of opioids associated with increased prescription for chronic pain [17]. In some European countries, though the prescription of opioids is not threatening, there is a trend towards increased prescription [18]. In most European countries, including Portugal, the therapeutic regimen of patients with chronic pain seeks to avoid opioid drugs [19,20]. Portugal is representative of the regions with less opioid consumption, staying under the 5%-mark, prevalence-wise [21,22,23]. This is mostly due to the negative impact of their side-effects, which motivate opioid-avoidant prescription practices [20,24,25]. The known side-effects of opioid drugs, such as obstipation, respiratory depression, tolerance and especially their addictive potential weight strongly against the decision of European clinicians to prescribe drugs of this class [26,27].

In what concerns the epidemiology of OIH in patients with chronic pain, it’s currently impossible to calculate the incidence or prevalence due to lack of substantial and comparable data [7,11]. The two largest multi-center clinician surveys were conducted in 2020, but each relied on very different methods to estimate the impact of OIH on public health. In the first study, OIH incidence was calculated based on the perceived number of OIH cases recalled to be seen in a physician’s lifetime of practice, divided by the mean number of years of active clinical practice among survey respondents [7]. In this study, OIH was estimated to occur in 6.8 cases per clinician practice-year [7]. In the second study, clinicians were questioned as to their perception of OIH prevalence among patients. In this study, most clinicians answered that 5% of their patients with chronic pain developed manifestations attributable to OIH [6].

#### 3.1.2. Clinical Studies

Until a decade ago, OIH development was only validated in normal human volunteers receiving acute-morphine infusions [4]. Recent studies, conducted in chronic pain patients, indicate the development of OIH in patients with chronic pain [13,28,29]. Although opioids are not used as first-line treatment, in the absence of effective or new pain drugs, opioids are still used in the treatment of several chronic pain conditions such as chronic post-surgical pain [30], fibromyalgia [31], low back pain [32] some neuropathic pain syndromes [33] and migraine [34]. Several studies report increases in perceived pain scores after increases in the prescribed doses of opioids in these chronic pain conditions [3,6,11,35]. In the following paragraphs we will review recent data presenting evidence of OIH upon use of opioids in the above-mentioned chronic pain conditions.

Table 1 summarizes the characteristics of relevant studies in the context of chronic opioid therapy performed in clinical populations with chronic pain treated with opioids. Several studies presented in Table 1 show that opioids produce either no analgesic effects [36,37] or increase pain symptoms [13,29] in patients with chronic pain. Opioid tapering to lower doses [28] or the administration of low doses of the opioid antagonist naltrexone [13,29,38] revealed successful analgesia (Table 1). The reversion of OIH by using adjuvant substances that interfere with MOR signaling, other than naltrexone, has also shown promising results. NMDA-MOR heterodimerization has been studied as an important mechanism underlying OIH. In that regard, ketamine, an inhibitor of NMDA-type calcium channels was shown to successfully reverse OIH by high-dose remifentanil [39]. In a different study, the addition of antagonists of the δ-opioid receptor (DOR) has also proven effective as DOR signaling was postulated to interfere with the activity of NMDA type Ca^2+^ channels as well [40]. Another study in Table 1 shows higher times to normalization of pain threshold levels in patients previously exposed to opioids compared to opioid-naïve patients [37]. This study argues in favor of a life-long exposure to opioids inducing OIH in a dose- and time- dependent manner. Whether or not OIH develops in a dose-dependent manner is not consensual [10,11]. The specificity of tested opioid substances, administration route and tested pain modality may also have to be considered [4] in addition to further contextual and individual variables [41].

The limited information available on physicians’ perceived relation between OIH onset and specific chronic pain syndromes favors the perspective that OIH onset isn’t related with any specific health condition being treated, instead it rather happens as a side-effect of opioid administration, independently of its justification [6]. An extensive review of individually reported patients’ cases with the diagnosis of OIH has been recently published [5]. Higher expression of clinical OIH was associated with high daily opioid doses, independent of the underlying condition, and therapeutically addressed through change of opioid analgesic, opioid cessation or use of non-opioid adjuvants, mainly ketamine or dexmedetomidine, which also resulted in the greatest reduction of administered opioid doses [5]. Additionally, many patients with chronic pain depend on strong analgesic therapies, which include opioids, to maintain quality-of-life. When used in such circumstances, and physicians diagnose a probable case of OIH, opioid switching, that is, change of the active opioid substance, reduction of opioid dose and the use of adjuvant substances have also been shown to counteract the molecular mechanisms of OIH [5,6,7,11].

It’s important to be aware, as well, to how opioids seem more easily able to promote patients’ satisfaction with care than they are to reduce actual pain intensity, when compared with non-opioid analgesics, in the context of non-cancer-related chronic pain care centers, as supported by several observational studies [22]. This may reflect the specific pain-masking effect of opioid substances, with associated cognitive conditioning coursing with subjective attribution of smaller importance to nociceptive inputs [11,42]. On the other hand, it’s important to note that opioids also modulate cognitive and affective/emotional brain processes, by affecting several key areas, including the amygdala, anterior cingulate gyrus, prefrontal cortex, thalami, and several brainstem nuclei [17,43,44]. An imaging study in patients with chronic pain showed reduced MOR availability associated with decreased pain-evoked activity in many of these areas [45] (Table 1). The authors suggest that these alterations contribute to affective pain dysregulation.

**Table 1:** Characteristics of relevant studies in the context of chronic opioid therapy: study type, setting, patients and tested substances.

Study Reference	Subjects		Study		Tested Substances			Outcome Comparison with Respective Controls	OIH or Tolerance	
	Total N	Pain Conditions (N)	Design	Test Group	Opioid Analgesia	Non-opioid Adjuvant	Control Group		Seen?	Reversible w/Adjuvant?
[36]	41	Fibromyalgia (n = 10)	RCT		“10 mg Morphine or 0.2 mg/mL Naloxone”	-	Placebo	MPT = (similarly ↑)	No.	-
		Rheumatoid Arthritis (n = 11)						MPT = (similarly ↑)	No.	-
[45]	18	Fibromyalgia (n = 18)	Intra-group analysis of MOR expression through fMRI	Patients with lower MOR expression	None (naïve)	-	Patients with higher MOR expression	PEA ↓ in antinociceptive nuclei and *nucleus accumbens*	No.	-
[38]	10	Fibromyalgia (n = 10)	RCT		None (naïve)	4.5 mg naltrexone	Placebo (no naltrexone)	MPT ↓ RPI ↓	No.	-
[29]	300	Fibromyalgia (n = 37) Opioid addiction (n = 69) Non-specified pain (n = 148)	Observational: cold pressor test after past morphine exposure	High lifetime-opioid exposure	Variable (lifetime estimate of morphine-equivalent exposure)	-	Healthy controls (n = 46);(Low lifetime-opioid exposure)	MPT ↑ RPI ↑	Yes.	Yes.
						Added low-dose naltrexone	No added low-dose naltrexone	MPT ↓ RPI ↓		
[13]	76	OIH (n = 55) Fibromyalgia (n = 21)	Open-label case series		Buprenorphine (single dose)	Added low-dose naltrexone	No added low-dose naltrexone	MPT ↓	Yes.	Yes.
[28]	7	chronic neuropathic pain (n = 7)	prospective observational study.	Planned opioid tapering	Variable (≥120 mg morphine equivalents/day)	-	The same patients, before opioid taper was applied	MPT ↓ RPI ↓	Yes.	-
[46]	103	Low back pain (n = 103)	RCT	sustained-release morphine	Morphine or Remifentanil	-	The same patients, before analgesia was applied	RPI ↓	Yes (OIH excluded).	-
[47]	21	Low back pain (n = 21)	prospective observational study.	10 kHz spinal cord stimulation	none	-	Standard (opioid-including) practice	TOC ↓ RPI ↓	?	-
[37]	98	Low back pain (n = 70)	RCT	Planned opioid tapering	Morphine?	-	Healthy subjects (n = 28)	TOC ↑ RPI ↑ MPT ↑	Yes	-

RPI = Reported pain intensity; MPT = quantitatively measured pain (inverse to measured threshold); PEA = pain-evoked brain activity; TOC = Total opioid consumption; ↑ = Value of outcome variable increased in the experimental group (relative to the control group); ↓ = Value of outcome variable decreased in the experimental group (relative to the control group).

### 3.2. OIH in Post-Operative Pain

#### 3.2.1. Trends in Opioid Analgesia in Surgery

Practices of opioid prescription in the surgical context depend much on local practices and vary in different regions of the world [48,49,50,51]. Fentanyl is one of the most widespread opioids for intraoperative analgesia, but not the only one, as sufentanil and remifentanil are also prominent examples [10,49]. In the post-surgical period, morphine is widely used for pain control: systems exist that allow for both physician- and patient- controlled administration of analgesia.

The use of opioids in the surgical context is particularly extensive in North America [52]. While the surgical environment may be considered closed and relatively controlled, the USA report a high incidence of opioid misuse and a growing body of literature supports that the introduction of opioid pharmacotherapy in the surgical context contributes to higher rates of future opioid misuse, exposing reasons ranging from withdrawal symptoms after patient discharge to easy accessibility to opioids due to spare drugs being stored in patient’s homes after completion of a prescribed analgesic regimen in the outpatient setting [53].

In recent years, research efforts are being made to expose the advantages of multimodal anesthesia and peri-surgical pharmacotherapy regimens, with focus on reducing the use of opioids and reducing associated morbidity and mortality, [54,55]. There is evidence reporting worse surgical outcomes with higher doses of opioid analgesia [56], however the discussion of the impact of opioids on surgical success rates is past the scope of this review, which is targeted only at objectives of pain control.

#### 3.2.2. Review of Clinical Evidence

In Table 2 we summarize the studies that directly show the occurrence of OIH or indirectly suggest OIH in the surgical context. Evidence of OIH gathered from meta-analysis in the surgical context is summarized on Table 3. The most significant evidence of OIH in the post-operative setting is found in adult opioid users. It is well established that preoperative opioid use constitute a risk factor for severe postoperative pain [57] and chronic postsurgical pain [30]. Even low dose preoperative morphine showed increased pain intensity following surgery [58]. ketamine, which disrupts the NMDA-MOR interaction associated with OIH, as discussed in the next section, has been suggested as potentially useful in the perioperative period in these patients [59,60]. A recent meta-analysis of nine RCTs in adult opioid users, with at least two weeks preoperative opioid intake, undergoing surgery showed that perioperative ketamine reduced cumulative mean opioid consumption by 97.3 mg (95%CI: −164.8 to −29.7) after 24 h and 186.4 mg (95%CI: −347.6 to −25.2) after 48 h [61] (Table 3).

In a meta-analysis covering a total of 1494 patient, intra-operative remifentanil was shown to be dose-dependently correlated with subsequent mechanical hyperalgesia [10] (Table 3). The higher the dose of opioids administered during surgical intervention the higher the required dose of opioids afterward, which one could argue to be a manifestation of tolerance, if it wasn’t for the accompanying decrease in pain threshold [10]. A consequential effect of hyperalgesia (pain sensitization was observed, and not simply a “similar response requiring a higher opioid dose” as would be the case for tolerance), relatable to the dose of opioids used, is evident in the literature, even if the term “OIH” was not explicitly used for it.

Most of the studies showing OIH after surgery involve the use of intraoperative remifentanil [62,63,64,65,66,67,68,69,70] (Table 2). In fact, remifentanil is the only opioid analgesic for which a clear clinical OIH-inducing property has been demonstrated by a meta-analysis (Table 3) [10]. Fentanyl at high doses also also cause OIH [71] (Table 2). The OIH-reversive effect of dexmedetomidine and esmolol, used as adjuvants, (Table 2) [65,70] illustrate the interaction between MOR and the α2- β2- adrenergic receptors, respectively. The efficacy observed for pregabalin, also used as an adjuvant (Table 2), does not share the same fundamental mechanisms as proven for neuropathic pain, which are based on the peripheral up-regulation of α_2_δ-1 subunits of voltage-gated calcium channels. Instead pregabalin likely exertss anti-OIH effects via the suppression of supraspinal serotoninergic descending pain facilitation pathways that are exacerbated in OIH [62,72].

**Table 2:** Characteristics of relevant studies in surgical contexts of analgesia: setting, tested substance, patients, and procedure.

Study Reference	Subjects		Tested Substances				Outcome Comparison with Respective Controls	OIH or Tolerance	
	N	Surgery	Intraoperative Opioid	Postoperative Opioid	Non-Opioid Adjuvant	Control		Seen?	Reversible w/Adjuvant?
[62] *	90	Laparoendoscopic single-site urologic	Remifentanil	Morphine	Single dose Pregabalin (300 mg)	No adjuvant pregabalin	TFD ↓ AMA ↓ RPI ↓ MPT ↓	Yes *	Yes
[63]	64	Major lung surgery	Remifentanil	Buprenorphine (25 μg h^−1^)	-	Postoperative morphine	TFD ↑ AMA ↓	Yes *	-
[73]	36	Minimally invasive lumbar decompression	None (opioid-free regimen)	Morphine	-	Standard practice: opioid-containing anesthesia	TOC ↓ RPI = LOS ↓	No	-
[74]	62	Posterior spinal fusion for adolescent idiopathic scoliosis (n = 37)	Remifentanil	Morphine	-	Intraoperative fentanyl, standard protocol (n = 25)	TOC ↓ RPI ↓ LOS =	Unlikely	-
[64]	40	Elective cardiac surgery	Remifentanil-controlled target dose (<dose)	Morphine	-	Remifentanil-continuous infusion (>dose)	AMA ↓ MPT ↓ RPI = TOC =	Yes	-
[65]	48	Thyroidectomy	Remifentanil	Morphine	Dexmedetomidine	No adjuvant dexmedetomidine	RPI ↓ MPT ↓ AMA ↓ TOC ↓	Yes *	Yes
[66]	40	Laparoscopic gynecologic surgery	Remifentanil	Morphine	Ketamine	No adjuvant ketamine	RPI ↓ TOC ↓	Yes *	Yes
[67]	47	Total thyroidectomy	Remifentanil	Morphine	Acetazolamide	No adjuvant acetazolamide	AMA = RPI = TOC = MPT = (↓ in 2 groups)	Yes *	No
[71]	56	Laparoscopic sleeve gastrectomy	Fentanyl, high dose (>dose)	Morphine	-	Intraoperative fentanyl, standard protocol (<dose)	TFD ↑	Yes	-
[68]	75	Laparoscopic gynecologic surgery	Remifentanil	Morphine	Ketamine	No adjuvant ketamine	MPT ↓ TOC ↓ RPI ↓	Yes *	Yes
[70]	60	Laparoscopic cholecystectomy	Remifentanil	Fentanyl	2 groups: (A) ketamine and (B) esmolol	No adjuvants (neither A nor B)	Similar in groups A & B: TOC ↓ RPI ↓	Yes *	Yes (in both groups)
[69]	54	Laparoscopic cholecystectomy	Remifentanil	Morphine	Ketamine (preoperative)	No adjuvant ketamine	RPI ↓	Yes	Yes

TOC = Total opioid consumption in postsurgical recovery; TFD = Time to first dose of postoperative morphine; LOS = Length of stay; AMA = Area of mechanical allodynia; RPI = Reported pain intensity after surgery; MPT = quantitatively measured pain (inverse to measured threshold); ↑ = Value of outcome variable increased in the experimental group (relative to the control group); ↓ = Value of outcome variable decreased in the experimental group (relative to the control group). * These studies didn’t include a control group to verify OIH induction, and only tested OIH reversion using the adjuvant substance. A degree of OIH was assumed to be present in all patients, and this assumption was in accordance with findings of increased pain intensity and manifestations of mechanical allodynia, in a manner that was dependent on opioid dose; this was compatible with the conclusions of the meta-analysis by Fletcher et al. [10].

**Table 3:** Evidence gathered from meta-analysis implicating OIH occurrence and treatment in surgical settings.

Study Reference	Substance Used	Outcomes	Outcome Analysis
[10]	Intra-surgical remifentanil.	RPI ↑	Mean differences (on a 100 cm visual analogue scale) for high intra-operative doses *vs* reference group: 9.4 cm (95% CI: 4.4, 14.5) at 1 h; 7.1 cm (95% CI: 2.8, 11.3) at 4 h; 3 cm (95% CI: 0.4, 5.6) at 24 h.
		TOC ↑	Higher postoperative morphine use after 24 h (Standardize mean difference: 0.7 (95% CI: 0.37, 1.02)
[39]	Peri-surgical ketamine.	TOC ↓	Reduction of postoperative opioid consumption: 8 mg morphine equivalents (95% CI 6 to 9); 19% from 42 mg consumed by participants given placebo-over 24 h 13 mg lower (95% CI 10 to 15); 19% from 67 mg with placebo-over 48 h
		TFD ↑	Increased the time for the first postoperative analgesic request by 54 min (95% CI 37 to 71 min) vs. 39 min with placebo
		RPI ↓ (at rest)	Reduction of pain at rest: by 5/100 mm on a visual analogue scale (95% CI 4 to 7; 19% lower from 26/100 mm with placebo-at 24 h by 5/100 mm (95% CI 3 to 7; 22% lower from 23/100 mm-at 48 h
		RPI ↓ (during movement)	Reduced pain during movement: by 6/100 mm, 14% lower from 42/100 mm-at 24 h by 6/100 mm, 16% lower from 37 mm-at 48 h
		AMA ↓	Reduced the area of postoperative hyperalgesia by 7 cm² (95% CI −11.9 to −2.2), compared with placebo
[50]	Opioid-free anesthesia.	RPI = (at rest)	Pain scores at rest at two postoperative hours were equivalent in the opioid-inclusive and opioid-free groups with a mean difference of 0.2 (95%CI: −0.2 to 0.5)
		LOS =	Similar length of stay in the recovery area, the mean difference 0.6 min (95%CI: −8.2 to 9.3)
[61]	Peri-surgical ketamine in chronic opioid users.	RPI ↓ (during movement)	Low-quality evidence that ketamine may slightly reduce postoperative pain during movement after 24 h (mean difference: −0.79; 95% CI: −1.22 to −0.36)
		TOC ↓	Reduction of cumulative mean opioid consumption: by 97.3 mg (95%CI: −164.8 to −29.7) after 24 h by 186.4 mg (95%CI: −347.6 to −25.2) after 48 h

TOC = Total opioid consumption in postsurgical recovery; TFD = Time to first dose of postoperative morphine; LOS = Length of stay; AMA = Area of mechanical allodynia; RPI = Reported pain intensity after surgery; ↑ = Value of outcome variable increased in the experimental group (relative to the control group); ↓ = Value of outcome variable decreased in the experimental group (relative to the control group).

### 3.3. Neurobiological Research on OIH in Acute and Chronic Non-Cancer Pain and Correlations between Bench and Bed-Side Findings

Opioids together with GABA and the monoamines serotonin and noradrenaline represent the main neurotransmitters involved in the modulation of nociceptive transmission from the primary sensory neurons, whose cell bodies are located within the dorsal root ganglia (DRG), to spinal dorsal horn neurons travelling to the brain. Opioids and GABA are released locally, by spinal interneurons, as well as by descending fibers originated in the brainstem, that also release serotonin and noradrenaline [44]. The most well characterized descending systems arise from the periaqueductal gray (PAG)-rostral ventromedial medulla (RVM) pathway, the locus coeruleus (LC) and the dorsal reticular nucleus (DRt) [44]. Descending serotonergic fibers, arise from the RVM, and play a fundamental role in descending pain facilitation. The LC is involved in descending pain inhibition through the release of noradrenaline acting on spinal α2-ARs, while the DRt facilitates nociceptive transmission through direct projections to the spinal dorsal horn [75,76,77]. OIH entails several cellular and molecular alterations within these neural systems. Inflammatory mediators released due to tissue damage together with neural-inflammatory crosstalk play a crucial role in OIH [2]. In the following paragraphs, we focus on the most relevant biological mechanisms for OIH, and their correlations with the clinical manifestations of OIH.

#### 3.3.1. Neuroinflammation

Spinal glial cells have been closely associated with OIH. In a rodent study, it was noted that the continuation of hyperalgesia induced by 10-days daily morphine administration depended on microglial cell reactivity and activation of the inapt immunity agent Toll-like receptor 4 (TLR4), meanwhile appointed as possible pharmacological target for OIH reversion [78,79]. The opioid antagonists naloxone and naltrexone cause inhibition of TLR4 [79]. In the acute context, TLR4 inhibition has been shown to increase effectiveness of acute opioid analgesia [80]. Human studies performed on patients with chronic pain, demonstrated improved clinical outcomes when small-dose opioid antagonists were used in conjunction with the primary MOR-acting analgesic, causing significantly less hyperalgesia-like manifestations after opioid administration [5,13,29]. Several studies point to a prominent role of microglia at the spinal cord in OIH. The M1 (proinflammatory)- and M2 (anti-inflammatory)-paradigm has been used to polarize microglial phenotypes according to their functional properties regarding inflammation. In the context of chronic morphine administration, in rodent models, the proportion of M2-polarized spinal microglia was shown to be increased in the spinal cord [81]. This increased of M2-microglia was interpreted as an effort to resolve the neuroinflammation caused by chronic morphine administration. In the context of chronic pain, morphine was shown to prolong neuropathic pain through the activation of NOD-like receptor protein 3 (NLRP3) inflammasomes, a signaling platform, expressed by spinal dorsal horn microglia [82]. More recently, the same research group showed that damage associated molecular patterns (DAMPs), high mobility group box 1 (HMGB1) and biglycan, persistently elevated in male rats during morphine-induced persistent sensitization, are causal NLRP3 activators [78]. A rodent study exploring the role of P2X purinoceptor receptors in OIH, found that OIH entails the upregulation of P2X receptor subtypes expression in DRGs [83]. Interestingly, morphine treatment results in persistent DAMP release via P2X7R and TLR4 signaling [78]. Therefore, NLRP3 inflammasomes at the spinal cord might be persistently activated by DAMPs signaling at these receptors [78]. The adenosine signaling further contributes to the perpetuation of this mechanism of neuroinflammation at the spinal cord in the context of OIH. Findings in rodents show that the A_3_ adenosine receptor (A_3_AR) signaling in the spinal dorsal horn is compromised in morphine-caused OIH and that the reduced A_3_AR activation culminates in NLRP3 inflammasome activation [84]. The results of this study further suggest the therapeutic utility of targeting the adenosine system by A_3_AR agonists as opioid adjuncts to avoid opioid side effects such as OIH [84].

Neuroinflammation is not an isolated event. Interaction between neuronal and glial cells is responsible for the phenomenon of hyperalgesic priming. This apparently protein kinasec Cε-mediated mechanism consists of increased neurons’ susceptibility to nociceptive transmission by exposure to inflammatory mediators, such as those released by adjacent activated glial cells. Neuronal TLR4 has a key role in hyperalgesic priming, concordant with the neuron’s reaction to inflammatory mediators. As mentioned above, low-dose opioid antagonists inhibit TLR4 activation and are reasonable anti-OIH substances in humans with chronic pain, however, the relative importance of glial vs. neuronal inhibition of TLR4 is not discernable in available literature, as trials have, so far, only addressed chronic pain conditions [13,29]. Despite this, consideration that morphine may prolong activation of NLRP3 inflammasome and promote OIH [78,79] may justify the rationale for low-dose naloxone/naltrexone efficacy in analgesia and their demonstrated anti-OIH effect in chronic pain settings [13,29]. Following morphine treatment leading to OIH and neuropathic-pain-like-behavior, observed chemokine alterations include upregulation of the stromal-derived factor-1, which is a ligand of CXCR4 receptor [85]. In the DRG, pronounced CXCR4 expression, found in satellite glial cells, and functional CXCR4 expression in DRG neurons was observed in chronic morphine-induced OIH, with the hyperalgic phenomenon being reversed with the intraperitoneal infusion of a CXCR4 antagonist [86]. CXCR4 antagonists have been demonstrated to successfully inhibit OIH in rodent models [87], as well as to inhibit signaling via the CC chemokine receptor type 1 (CCR1) [88]. Another recent study regarding the complex mediation between spinal neuroinflammation and opioid related nociception indicates that the immune checkpoint inhibitor programmed cell death protein-1 (PD-1) as another key element. The loss of PD-1 prolongs OIH in rodents and non-human primates [89] and seems to impair inhibitory GABA signaling provoking chronic pain phenotypes in knockout rodents [90]. Despite these findings, as far as we know, no clinical trial in humans has yet explored these specific interactions as potential therapeutic targets.

#### 3.3.2. Cellular and Molecular Alterations Involved in OIH

Long term potentiation (LTP) at C fibers synapses represents a cellular model for pain amplification (hyperalgesia) [91]. LTP is dependent on glutamatergic stimulation via NMDA receptors and this has also been shown to be mediated by activated glial cells, and signaling via neurotrophic stimulating factors (like BDNF) and mediator cytokines (like 1L-1β and TNF-α) [2,92]. Antinociceptive actions mediated through GABA receptors on neurons were posed to be impaired and therefore to contribute further to LTP in hyperalgic signaling, enhancing perpetuation of OIH [93,94]. The impairment of GABA receptors in these conditions, while experimentally achieved through induction of inhibitory neuronal KCC2 channels [2,95], was not shown to be clinically applicable in acute OIH using acetazolamide, a pharmacological inhibitor of carbonic anhydrase in clinical use [67,96]. In an RCT involving patients under surgical analgesia using remifentanil, concomitantly revealing hyperalgic manifestations compatible with OIH, acetazolamide was unsuccessful in reverting these disturbances through the additional use of this substance [67], unlike other substances applied in similar conditions as mentioned in Table 2.

MOR is a transmembrane G-protein coupled receptor encoded by the OPRM1 gene through which opioid drugs produce analgesia as well as OIH [97]. It is expressed at high levels in primary afferents, the spinal dorsal horn and in the aforementioned brainstem structures [98]. OIH has been shown to be the result of maladaptations in downstream MOR signaling that lead to increased neuronal excitability. These alterations in MOR signaling pathways include the switching of MOR coupling from an inhibitory G-protein to a stimulatory G-protein, abnormally promoting phosphorylation and intracellular activity dependent on PKA-dependent pathways, which favor neurotransmission in the afferent neurons of the nociceptive pathways [44,99,100]. The inhibitory-to-stimulatory modification in MOR signaling has also been demonstrated in the DRt, in experimental models of OIH due to chronic morphine treatment while DRt inactivation with targeted lidocaine injections successfully reversed OIH experimentally [97].

Another cellular change triggered by MOR is the altered profile of MOR heterodimerization. MOR has been shown to form protein dimers with a list of other receptors that’s not yet fully described, which includes for example other opioid receptors such as DOR, the NMDA and cholecystokinin (CCK) receptors and cytokine receptors such as CX3CL1 and CXCR4 [101,102]. Molecular activation/inhibition of each receptor within the heterodimer affects the signaling of one another [102]. An additionally occurring factor is heterologous desensitization: a phenomenon in which receptors are withdrawn from the cell membrane due to activation of their paired dimer [102]. Therefore, reinforced justification arises for the ability of non-opioid adjuvants to interfere with opioid signaling through conditioning of MOR signaling. Further research on MOR heterodimerization may contribute to the development of more alternatives to these adjuvant molecules, with relevant biological and clinical modulation capacity upon opioid signaling and pain processing [103]. For instance, the CCK antagonists RSA 504 and RSA 601 enhance opioid analgesia and diminish sensitivity to mechanical and thermal stimuli in a rodent model of neuropathic pain [104,105]. Conversely, CXCR4 agonists impaired opioid analgesia, by diminishing MOR signaling and causing heterologous desensitization at the level of MOR-CXCR4 heterodimers in the PAG. Similar results were observed regarding MOR-CCR5 heteromers [102]. A different form of crosstalk has been found between MOR and TRPV1, which is due to a β-arrestin sequestrating action induced by MOR stimulation [106].

Another phenomenon that’s of relevant consideration is that the OPRM1 gene undergoes several processes of alternative pre-mRNA splicing, resulting in a pool of slightly different MOR-like protein structures which constitute a profile of isoforms of the µ opioid receptor [98,107]. Within this pool of isoforms, 3 major groups are identifiable, and biologically conserved in mice, rats, and humans. The OPRM1 gene has two open reading frames, one starting on exon 1 and the other on exon 11. Most isoforms with modified intracellular C-terminals derive from beginning of transcription at exon 1 and contain 7 transmembrane domains (7TM), the same as the native/canonical receptor; conversely, most isoforms with modified extracellular N-terminals derive from beginning of transcription at exon 11 and contain only 6 transmembrane domains (6TM) [98,107]. A chaperone protein exists, consisting only of exons 1 and 4, functioning in the endoplasmic reticulum, leading to increased expression of the canonical MOR-1 and enhancing morphine analgesia [98,108]. From the total population of MOR proteins in a cell, the proportion of alternative isoforms is not insignificant [98,109,110]. It’s possible to differentiate the intracellular signaling promoted by 7TM and 6TM isoforms, in favor of inhibitory G- protein coupling in 7TMs and stimulatory-G-protein coupling in 6TMs [110]. It was postulated that the profile of MOR isoforms present in mice, rats and human neurons differs from the respective physiological normal under established OIH conditions, something that accompanies alterations in signal transduction following opioid stimulation in favor of pro-nociceptive transmission [110]. In fact, mu agonists can induce differential signaling through multiple 7TM isoforms splice variants [107]. Opioid tolerance may be impacted by isoforms as well, since alternative splicing at the 3′ end impacts the protein’s intracellular tail, potentially weakening intracellular signal transduction, as well as manipulating membrane-to-endosome molecular traffic, resulting in reduced cellular receptivity to opioid stimulation [98,107,110].

It is rational to conceive connections between the profile of isoforms and the patterns of heterodimerization, as different isoforms adopt different conformations, with differently positioned outlying exons, justifying isoform-specific predispositions towards dimerization with other membrane proteins, such as other GPCRs. This has been demonstrated for the 6TM isoform mMOR-1K and the NMDA receptor, with which the NMDA receptor may form a stronger bond than with the canonical MOR [102,111]. Therefore, when considering the molecular interaction between NMDA and MOR-1K, morphine stimulation reinforces NMDA-derived effects, which are of neuronal activation, contrary to analgesia, in the case of the nociceptive pathways [111]. Indeed, the interaction between MOR and the NMDA receptor has been documented before, at the PAG level, but presented as a mechanism to explain opioid tolerance [112]; however, since the effect of the opioid is to promote pain signaling through increased neuronal activity, via NMDA activation through the calmodulin-dependent protein kinase (CAMKII) pathway [102,112], the resulting hyperalgesia in this case is opioid-induced, and not due to lack of opioid receptor stimulation, constituting a phenomenon of OIH, by definition. Additionally, individuals that show clinical OIH may have been prone to enhanced manifestation of this interaction because of previous opioid exposure modifying the MOR-isoform profile itself [11,61]. Additionally, Zhang and collaborators contributed to the consideration of genetic variability as a determinant of the individual-specific sum of impact from the mentioned molecular signaling pathways, and therefore a determinant for opioid-response and observable clinical variability, as the team demonstrated OPRM1 gene polymorphisms influenced individual women’s response to fentanyl analgesia after lower segment caesarean section surgeries [113].

### 3.4. OIH in Cancer Pain and Challenges in the Duality between Suffering (“Pathos”) and Pathology

Both nociceptive and neuropathic components of pain are present in the setting of chronic oncological pain. This context is further complicated by the complex influences that affective and cognitive valences have on pain modulation, since cancer is normally a harsh reality in the life of suffering patients. In what specifically concerns OIH, only a few case reports have been published where oncological patients experienced augmented pain after an increase in prescribed opioid dose, with analgesic intent [114]. Nevertheless, a large proportion of cancer patients suffer with pain during treatment [115]. Pain may arise from the neoplasia itself, its physical influence on surrounding tissues (mostly nociceptive in nature), due to cancer-promoted inflammation, caused by treatments, such as chemotherapy and radiotherapy, as well as acute distress from surgical interventions, which may also include a neuropathic component due to nerve damage, all summed with variable degrees of opioidergic dysregulation induced by therapy.

The nociceptive and neuropathic components of pain in cancer patients likely overlap with OIH mechanisms. This is in line with the fact that a significant portion of cancer patients does not respond to opioid therapy and that pain in these patients becomes manageable when opioids are given with ketamine as an adjuvant [116]. In addition to this, in 2019, a RCT was published revealing safety when using intravenous lidocaine during oncological surgery of head and neck. Lidocaine possesses both local anesthesic actions, through the blockade of neuronal activity and anti-inflammatory interference. Lidocaine reduced overall post-surgical consumption of opioid analgesics and long-term reduction in analgesic needs [117]. Regarding treatment such as chemotherapy, we have shown that chemotherapy-induces neuropathic pain which entails alterations in the descending pain modulatory system, such as a progressive exhaustion of the descending noradrenergic inhibition [118] and an engagement of the facilitatory influences of the descending serotonergic system [119]. Strikingly, such alterations in the descending pain modulatory, constitute underlying mechanisms of OIH [72,120]. Thus, cancer patients are likely at risk of developing OIH further aggravated by chemotherapy. Treating pain in cancer patients with opioids might be counter-productive unless their opioid regimen is carefully monitored and appropriate measures to counter the molecular mechanisms of OIH are preventively implemented as discussed above. The relevance of tailored pain therapy in these patients is therefore further reinforced by the likely occurrence of clinical manifestations of OIH.

## 4. Conclusions

The clinical data reviewed comprised clinical evidence of OIH development in chronic pain and in post-surgical settings. The vulnerability to OIH is due to several factors, including an imbalance of the downstream inhibitory and excitatory MOR signaling towards a neuroexcitatory signaling as shown at the molecular level by recent basic studies. This correlates well with the clinical data showing possible degrees of neuroexcitation, from subclinical hyperalgesia (conserving the overall analgesic effect) to pathological hyperalgesia, as described in the published clinical cases of OIH [5], where this phenomenon, when intensely manifested, becomes the main problem for the patient, acting as a true “disease”. Considering the diagnostic approaches to such “disease”, as well as the targeted treatment it requires [6], the OIH phenomenon is understatedly clinically relevant.

The importance of OIH is reinforced by the increasing trends in opioid prescription worldwide [23,52], since it may originate as a side-effect of therapy, possibly in concordant association with lifelong cumulative exposure to opioids, reinforced by high-dose acute exposure [53,121]. In addition, it also may depend on factors conditioning the magnitude of the specific molecular interactions, such as the pharmacodynamic profile of the different opioid drugs and the inter-individual variations in cellular and molecular neurobiology [102], as is natural among living organisms. This is concordant with the successful application of opioid rotation, opioid cessation and select non-opioid adjuvant drugs that target OIH-specific mechanisms as therapeutic measures in clinical cases of OIH [5,6,7].

The degree to which the importance of OIH is understated is, however, still unknown, and a double-edged problem: while data is still missing regarding the estimation of overall OIH incidence, due to lack of robust epidemiological studies, its symptomatic similarity with other phenomena, such as opioid tolerance, and general ignorance of its existence among clinicians cause OIH to pass unnoticed, while easily misdiagnosed with other causes of therapeutic failure, to the harm of the patient [6,8,27]. Investing on medical education, regarding OIH’s clinical and biologic contexts, pathophysiology, and clinical features is therefore the way to allow for less OIH-related analgesic failures to pass misdiagnosed and mistreated. Finally, acknowledge unequivocally OIH in the clinic will undoubtedly also contribute to promote the search for improved opioid drugs for medical care of pain.
